# *In vitro* Evaluation of Medihoney Antibacterial Wound Gel as an Anti-biofilm Agent Against Ventricular Assist Device Driveline Infections

**DOI:** 10.3389/fmicb.2020.605608

**Published:** 2020-11-23

**Authors:** Yue Qu, David McGiffin, Christina Kure, Janelle McLean, Courtney Duncan, Anton Y. Peleg

**Affiliations:** ^1^Infection and Immunity Theme, Monash Biomedicine Discovery Institute, Department of Microbiology, Monash University, Clayton, VIC, Australia; ^2^Department of Infectious Diseases, The Alfred Hospital and Central Clinical School, Monash University, Melbourne, VIC, Australia; ^3^Department of Cardiothoracic Surgery, The Alfred Hospital and Monash University, Melbourne, VIC, Australia; ^4^Department of Medicine, Central Clinical School, Monash University, Melbourne, VIC, Australia; ^5^Transplant Services, The Alfred Hospital and Monash University, Melbourne, VIC, Australia

**Keywords:** Medihoney Antibacterial Wound Gel, anti-biofilm, methylglyoxal, ventricular assistant device, driveline infections

## Abstract

**Objectives:** In adult ventricular assist device (VAD) programs in Australian hospitals, Medihoney Antibacterial Wound Gel (MAWG) is routinely used at the skin exit-site of VAD drivelines to prevent infections; however, its effectiveness remains unclear. Our aim was to assess antimicrobial activity of Medihoney wound gel, using *in vitro* models that mimic clinical biofilms grown at the driveline exit-site.

**Methods:** Antimicrobial susceptibility testing of MAWG was performed for 24 clinical isolates grown under planktonic conditions, and four representative strains grown as biofilms. Different antimicrobial mechanisms of MAWG were assessed respectively for their relative contribution to its anti-biofilm activity. A colony biofilm assay and a drip-flow biofilm reactor assay mimicking the driveline exit-site environment were used to evaluate the activity of MAWG against biofilm growth at the driveline exit-site.

**Results:** MAWG demonstrated species-specific activity against planktonic cultures [minimum inhibitory concentrations (MICs), 5–20% weight/volume (W/V) for *Staphylococcus* species, 20–>40% (W/V) for *Pseudomonas aeruginosa* and *Candida* species]. Higher concentrations [MICs, 30–>80% (W/V)] were able to inhibit biofilm growth, but failed to eradicate pre-established biofilms. The anti-biofilm properties of MAWG were multi-faceted, with the often-advertised “active” ingredient methylglyoxal (MGO) playing a less important role. The colony biofilm assay and the drip-flow biofilm reactor assay suggested that MAWG was unable to kill biofilms pre-established in a driveline exit-site environment, or effectively prevent planktonic cells from forming adherent monolayers and further developing mature biofilms.

**Conclusion:** Our work suggests a suboptimal effectiveness of MAWG in preventing driveline infections due to biofilm development.

## Introduction

Medical-grade honeys have been used for the treatment of infection in chronic wounds and persistent diabetic ulcers (Soffer, 1976; Dunwoody and Acton, 2008). They have also been used for prophylactic indications such as prevention of peritoneal dialysis catheter exit-site infection (Forbes et al., 2016). Documented merits of honey as an antimicrobial agent include effectiveness against both planktonic cultures and biofilms (Lu et al., 2019), activity against multi-drug resistant microorganisms (Tirado et al., 2014), broad-spectrum antimicrobial activities (Israili, 2014), strain- and antibiotic-specific synergy with conventional antibiotics (Liu et al., 2017; Hayes et al., 2018), and a reported low risk of developing antimicrobial resistance (Lu et al., 2014). Antimicrobial and anti-biofilm effects of medical-grade honey have been attributed to various factors working either singularly or synergistically, including the production of hydrogen peroxide, the presence of specific antimicrobial agents, such as flavonoids, bee peptides and phenolic compounds [methylglyoxal (MGO)], special physiochemical properties including a low pH and exertion of high osmotic pressure, and its impact on the infection environment, such as desiccation of the wound (Osato et al., 1999; Israili, 2014; Cokcetin et al., 2016; Sowa et al., 2017).

Medihoney Antibacterial Wound Gel (MAWG, Comvita Ltd.) has been advertised by the manufacturer to be effective for all minor wounds including burns, cuts, grazes, and ulcers. Several *in vitro* studies have found that its main component, Manuka-type honey, is highly effective against bacterial biofilms (Cooper et al., 2014; Lu et al., 2014, 2019). As a consequence, MAWG has been used in all four Australian hospitals that perform adult ventricular assist device (VAD) implantation to prevent driveline infections by application at the driveline exit-site. Large randomized controlled trials, however, report only marginal effects of MAWG in preventing device-related infection in patients with percutaneous medical devices when compared with standard care with or without additional prophylaxis (Johnson et al., 2014; Zhang et al., 2015). The discrepancy between the *in vitro* efficacy of Manuka-type honey and *in vivo* efficacy of MAWG against biofilms may be partially due to the use of over-simplified microplate-based biofilm assays by many other *in vitro* studies (Cooper et al., 2014; Hammond et al., 2014; Lu et al., 2019). Microplate-based biofilm assays often neglect the impact of the clinical environment and might not adequately reflect infections at the unique skin exit-site of percutaneous medical devices (Buhmann et al., 2016).

The purpose of this study was to assess the antimicrobial efficacy of MAWG against biofilms causing VAD driveline infections, using *in vitro* assays that closely mimic driveline exit-site environments. The results of the study might determine if there is sufficient experimental support for the clinical use of MAWG to prevent driveline infections.

## Materials and Methods

### Medihoney Antibacterial Wound Gel, Other Media, and Drivelines

Medihoney Antibacterial Wound Gel™ (simplified as MAWG for this study, Comvita Australia Pty Ltd.) was used for this study. This licensed commercial product is specifically formulated combining 80% Medihoney™ Antibacterial Honey derived from the *Leptospermum scoparium* plant in New Zealand and 20% natural waxes and oils. MAWG solution was prepared by dissolving the gel into a standard microbial growth medium such as Muller-Hinton broth (MHB; Oxoid, Hampshire, UK) or Roswell Park Memorial Institute 1640 medium (RPMI 1640; Oxoid, Hampshire, UK) to reach concentrations of 0–80% [weight/volume (w/v); increments of 10%, equivalent to Medihoney™ Antibacterial Honey of 0–64% with increments of 8%]. To assess the contribution of different antimicrobial mechanisms of MAWG to its anti-biofilm properties, other media preparations were used. Mixed sugar solution comprising 45% glucose (w/v), 48% fructose, and 1% sucrose was prepared as described by others (Liu et al., 2014). This mixed sugar solution has the same osmolarity as pure Manuka honey and was diluted to match that of MAWG (containing 80% Medihoney Antibacterial Honey). MGO solution (40% in H_2_O) was purchased from Sigma Australia and was further diluted into the growth medium to a concentration the same as that found in MAWG (MGO: 620 mg/kg = 776 mg/kg × 80%; Liu et al., 2014; Lu et al., 2014). Microbiological growth media of different pH (pH = 7.0, 6.0, 5.0, and 4.0) were prepared by adding 5 M hydrogen chloride or sodium hydroxide. HeartMate III drivelines were provided by Abbott Medical, United States and were used for driveline biofilm experiments. Driveline silicone tubes (smooth section) were cut, and then transected into pieces of ~3 × 5 mm^2^. Prior to use in each experiment, the cut-out driveline sections were sterilized with ethylene oxide (Steritech, VIC, Australia).

### Microbial Strains

Twenty clinical isolates and four reference strains from three microbial genera frequently causing VAD driveline and other medical device related infections were selected for this study, including coagulase-negative *staphylococci*, *Staphylococcus aureus*, *Pseudomonas aeruginosa*, and *Candida* spp. (Table 1; Qu et al., 2010a, 2016, 2020; Wu et al., 2019). The clinical reference strains included *Staphylococcus epidermidis* RP62A (ATCC35984), *S. aureus* ATCC25923, *P. aeruginosa* PAO1, and *Candida albicans* SC5314. These reference strains were used to study the anti-biofilm efficacy of MAWG as they are well-known biofilm producers, they were representative of the clinical isolates in their sensitivity to MAWG under planktonic conditions, and they have been widely used in other *in vitro* studies, and parallel comparisons were possible.

**Table 1:** Antimicrobial activity of Medihoney Antibacterial Wound Gel (MAWG) against clinical isolates grown as planktonic cultures.^1^

Microbial species	Source	References	Antimicrobial activities	
			MIC^2^	MBC/MFC^3^
***Staphylococcus aureus***				
ATCC25923	Reference strain	Qu et al., 2020	10%	20%
APS 18	The Alfred Hospital	Unpublished	10%	20%
APS 19	The Alfred Hospital	Unpublished	20%	40%
APS 27	The Alfred Hospital	Unpublished	20%	20%
APS 28	The Alfred Hospital	Unpublished	10%	20%
APS 29	The Alfred Hospital	Unpublished	20%	20%
***CoNS***				
*Staphylococcus epidermidis* RP62A	Reference strain	Qu et al., 2020	20%	20%
*Staphylococcus epidermidis* RCH3	RCH^4^, Melbourne	Qu et al., 2010a	5%	10%
*Staphylococcus epidermidis* RCH 5	RCH, Melbourne	Qu et al., 2010a	10%	20%
*Staphylococcus capitis* RCH 6	RCH, Melbourne	Qu et al., 2010a	10%	20%
*Staphylococcus epidermidis* RCH 7	RCH, Melbourne	Qu et al., 2010a	20%	20%
*Staphylococcus epidermidis* RCH 12	RCH, Melbourne	Qu et al., 2010a	10%	10%
***Pseudomonas aeruginosa***				
PAO1	Reference strain	Qu et al., 2020	40%	40%
A0064	The Alfred Hospital	Unpublished	40%	40%
B0021	The Alfred Hospital	Unpublished	40%	40%
D0108	The Alfred Hospital	Unpublished	40%	40%
L0024	The Alfred Hospital	Unpublished	20%	20%
E0033	The Alfred Hospital	Unpublished	40%	40%
***Candida spp.***				
*Candida albicans* SC5314	Reference strain	Qu et al., 2020	20%	40%
*Candida albicans* APY49	The Alfred Hospital	Qu et al., 2016	40%	40%
*Candida albicans* VVC2	WMU^5^	Wu et al., 2019	40%	40%
*Candida albicans* VVC4	WMU	Wu et al., 2019	40%	40%
*Candida glabrata*	The Alfred Hospital	Unpublished	>40%	>40%
*Candida parapsilosis*	The Alfred Hospital	Unpublished	>40%	>40%

1 Antimicrobial activity of MAWG against clinical isolates was determined using broth microdilution assays, and the results were determined by viable counts, instead of examining turbidity or optical density.

2 MIC, minimum inhibitory concentration.

3 MBC/MFC, minimum bactericidal concentration/minimum fungicidal concentration.

4 RCH, The Royal Children’s hospital.

5 Wenzhou Medical University, China.

### Antimicrobial Activity of MAWG Against Planktonic Cells

Antimicrobial activity of MAWG against all 24 clinical and reference strains were evaluated by determining the minimum inhibitory concentrations (MICs) and minimum bactericidal or fungicidal concentrations (MBCs/MFCs), following Clinical and Laboratory Standards Institute (CLSI) guidelines with modification (Clinical and Laboratory Standards Institute, 2012, 2017). Viable counts were performed before and after overnight incubation, replacing turbidity-based growth assessment because of the cloudiness of MAWG solutions after incubation. Four concentrations of MAWG solutions (5, 10, 20, and 40%) were tested. MICs refer to the lowest concentration of MAWG at which no increase in microbial density was observed. The minimum concentrations of MAWG that reduced bacterial numbers by at least 3 log (99.9%) or fungal density by at least 1 log (90%) were defined as MBCs and MFCs. Three biological repeats in triplicates were carried out to determine the MIC, MBC, and MFC.

### Antimicrobial Activity of MAWG and Its Key Components Against Biofilms

Although microplate-based biofilm assays are not the best *in vitro* model to study driveline infections, they are ideal for quantitative examination of concentration-dependent effect of antimicrobials on inhibiting or killing biofilms. Single biofilms of *S. aureus* ATCC 25923, *S. epidermidis* RP62A, *P. aeruginosa* PAO1, and *C. albicans* SC5314 were set-up in 96-well microplates as previously described (Qu et al., 2016). Two hundred microliters of freshly prepared solutions, including that of MAWG, mixed sugar solution, and MGO in MHB or RPMI-1640 at increment concentrations were added into each microwell and treatments lasted for 24 h. Two anti-biofilm activity endpoints were assessed (Macia et al., 2014). Biofilm MICs (BMIC_50_) referred to the lowest concentration of agents that inhibit biofilm growth by 50%, as determined by 2,3-bis-(2-methoxy-4-nitro-5-sulfophenyl)-2H-tetrazolium-5-carboxanilide (XTT) readings (see below). Minimum biofilm eradication concentration (MBEC) referred to the lowest concentration that led to complete eradication of viable cells embedded in biofilms and were determined as described previously (Qu et al., 2010b).

### Biofilm XTT Assay

The XTT assay was adopted to assess the viability of biofilm cells after antimicrobial treatment (Qu et al., 2016). After treating established biofilms with MAWG solution, mixed sugar solution, and MGO at different concentrations, the suspensions were removed, and the microwells were washed twice with phosphate-buffered saline (PBS). Two hundred microliters of XTT solution (0.5 mg/ml) was added into each microwell, and the microplate was incubated at 37°C in the dark for 2 h. One hundred microliters of XTT solution was then transferred to a new microplate and read at OD_492_. The ratio of cell survival (OD_492_ after antimicrobial treatment) relative to antimicrobial-free culture (OD_492_ of the drug-free biofilms × 100) was calculated. Biofilm reduction was calculated as (1-cell survival%). The experiment was carried out in three biological repeats in triplicate.

### Agar Colony Biofilm Assay

The colony biofilm assay replicates some of the environmental conditions required for biofilm growth on a “relatively dry” wound bed of the driveline exit-site, by allowing microorganisms to grow on a filter membrane supplied with nutrients and oxygen but little shear stress (Hammond et al., 2011). In short, overnight microbial cultures were harvested by centrifuge, washed twice with PBS, and resuspended in MHB (for *S. epidermidis*, *S. aureus*, and *P. aeruginosa*, OD_600_ = 0.1), or RPMI 1640 (for *C. albicans*, OD_600_ = 1.0). Around 100 μl of each microbial suspension was seeded on sterile nitrocellulose filter membranes (diameter, 25 mm; pore size, 0.22 μm, Merck Millipore Ltd.,). The membranes were transferred onto either Muller-Hinton agar (MHA) or RPMI 1640 agar plates in a humid chamber and were incubated at 37°C for 2 h to grow early adherent monolayers or 24 h for mature biofilms (Merritt et al., 2005). Our preliminary scanning electron microscopy (SEM) assay suggested that 2 h incubation resulted in the attachment of a single layer of microorganisms to the filter membrane and 24 h incubation led to the growth of clusters of cells embedded in extracellular polymeric substances (EPSs). The filter membrane with early adherent monolayers or mature biofilms was completely covered with another filter membrane infused with 0.2 g of MAWG. This was to mimic the clinical application of MAWG in combination with wound dressings. The treatment lasted for 24 h. Both filter membranes were placed in a 15 ml falcon tube containing 5 ml of PBS and were sonicated for 10 min using a sonication bath (42 kHz, Branson 1510), followed by vortex at the highest speed for 2 min (30'' × 4). The suspensions were serially diluted and plated on nutrient agar plates or yeast peptone dextrose (YPD) agar plates for viable counts. The experiment was carried out in three biological repeats in duplicate.

### Microbial Adherence Assay and Drip-Flow Biofilm Assay Using Clinical Drivelines

To assess the effectiveness of MAWG in preventing planktonic cultures from growing early adherent monolayers on driveline materials, MAWG was applied on the surface of driveline cutouts and a microbial adherence assay was carried out (Qu et al., 2020). A drip-flow biofilm reactor was then used to evaluate the effectiveness of MAWG in preventing adherent monolayer on drivelines from further developing into mature biofilms (Goeres et al., 2009; Qu et al., 2020). This drip-flow biofilm reactor mimics a “wet” driveline exit-site environment by providing continuous flow of oxygen and nutrients and grows biofilms under low shear at the air-liquid interface (Goeres et al., 2009). Driveline cut-outs with attached microorganisms, prepared in the microbial adherence assay, were placed on the absorbent pads (25 mm, Millipore, Billerica, MA) with MAWG infused in the biofilm incubation chamber. Around 10% TSB as growth media were pumped through the system at 5 ml/h/channel. Biofilms were allowed to grow for 72 h at room temperature. The samples were washed three times with PBS, and were quantitatively analyzed for CFUs. The experiment was carried out in three biological repeats in duplicate.

### Statistical Analyses

One-way ANOVA test or a non-parametric Mann–Whitney method (depending on the data distribution) was performed to analyze differences in biofilm formation under different conditions, using Minitab 16 for Windows (Pennsylvania State University, United States) and a significance level of 0.05.

## Results

### Antimicrobial Activity of MAWG Against Planktonic and Biofilm Microorganisms Grown in 96-Well Microplates

Twenty-four clinically relevant isolates from three microbial genera were tested for their sensitivity to MAWG. Both *S. aureus* and coagulase-negative staphylococci were sensitive to MAWG when grown as planktonic cultures, with most isolates having MICs and MBCs of 10–20% (Table 1). *P. aeruginosa* and *Candida* spp. showed relatively higher resistance to MAWG, with MICs and MBCs/MFCs of 20–40% or even higher. To assess the efficacy of MAWG against microbial biofilms, we tested the BMIC_50_. MAWG at concentrations of 30, 50, >80, and 30% (W/V) were needed to inhibit biofilm growth by 50% for *S. aureus*, *S. epidermidis*, *P. aeruginosa*, and *C. albicans*, respectively (Table 2, see BMIC_50_). MAWG at the highest concentration used in this study [80% (W/V)], was unable to fully eradicate mature biofilms pre-formed by any of these microorganisms (Table 2, see MBECs).

**Table 2:** Susceptibility of microplate-based biofilms to MAWG and its active components.

	*Staphylococcus aureus* ATCC 25923		*Staphylococcus epidermidis* RP62A		*Pseudomonas aeruginosa* PAO1		*Candida albicans* SC5314	
	MBIC_50_	MBEC	MBIC_50_	MBEC	MBIC_50_	MBEC	MBIC_50_	MBEC
MAWG	30%	>80%	50%	>80%	>80%	>80%	30%	>80%
Mixed sugar	>80%	>80%	70%	>80%	>80%	>80%	70%	>80%
MGO	>80%	>80%	>80%	>80%	>80%	>80%	>80%	>80%

The biofilm MIC (BMIC_50_) refers to the lowest concentration of antimicrobials that is resulted in a 50% reduction of biofilm growth. Minimum biofilm eradication concentration (MBEC) refers to the lowest concentration of antimicrobial agents that completely kill embedded biofilm cells, showing no visible growth in the recovery medium used to collect biofilm cells after revival (Macia et al., 2014).

### MGO Plays a Less Important Role in the Multi-Faceted Anti-biofilm Activity of MAWG

**Figure 1:**
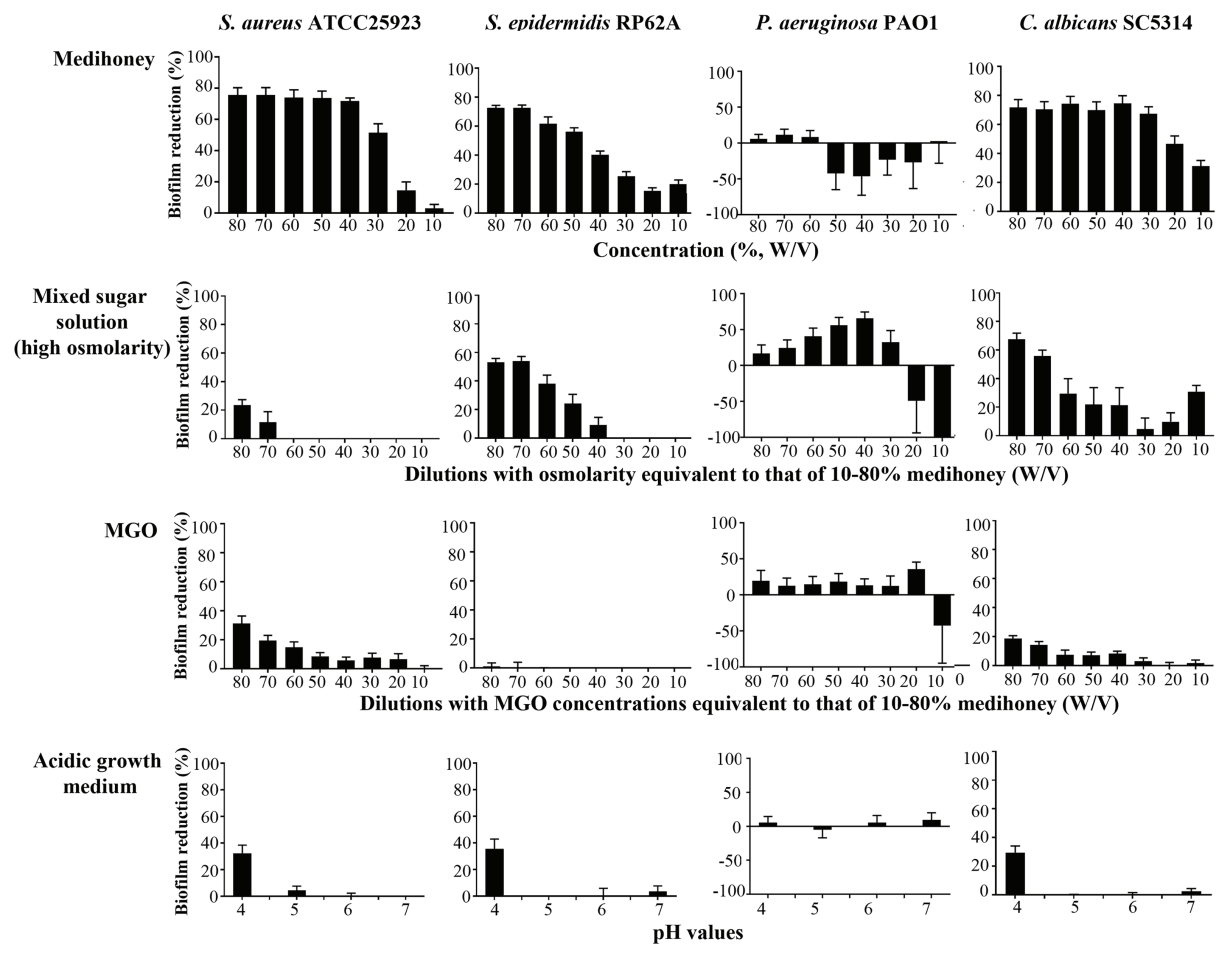
Assessment of the anti-biofilm activity of individual components of MAWG using microplate-based biofilms. Biofilms were established in 96-well microplates and were treated with MAWG solution, mixed sugar solution, methylglyoxal (MGO) solution at different concentrations, and growth media prepared at different pH. 2,3-bis-(2-methoxy-4-nitro-5-sulfophenyl)-2H-tetrazolium-5-carboxanilide (XTT) was used to detect the percentage of survivor biofilm cells after the treatment relative to untreated control. XTT readings at OD_492_ for untreated control biofilms were as below: *S. aureus* ATCC 25923, 0.94 ± 0.09 (mean ± SD); *S. epidermidis* RP62A, 0.89 ± 0.22; *P. aeruginosa* PA01, 0.25 ± 0.06, and *C. albicans*, 2.50 ± 0.50. The ratio of cell survival (OD_492_ after antimicrobial treatment) relative to antimicrobial-free culture (OD_492_ of the drug-free biofilms × 100) was calculated. Biofilm reduction was calculated as (1-cell survival%). Error bars indicate the standard error of the mean.

To assess the contribution of the individual antimicrobial mechanisms of MAWG to its overall anti-biofilm properties, BMIC_50_ and MBECs of MGO and a mixed sugar solution with high-osmolarity were determined, respectively (Table 2). For all pathogens, MGO showed a minor effect on biofilms, with the highest concentration (equivalent to 496 mg/kg) used in this study being unable to inhibit biofilm growth by 50% (Table 2). We then quantitated the actual viable cell reduction in biofilms achieved by varying concentrations of MGO (Figure 1). MGO at a concentration equivalent to that found in 80% (W/V) MAWG (MGO: 496 mg/kg) reduced the viability of biofilms of *S. aureus* by up to 30%, and by up 20% for *C. albicans* and *P. aeruginosa* (Figure 1). No evident activity of MGO was seen for *S. epidermidis* cells embedded in biofilms. In contrast to MAWG, mixed sugar solution conferring the high osmolarity had impaired activity in inhibiting biofilm growth, showing higher BMIC_50_ for *S. aureus*, *S. epidermidis*, and *C. albicans* (Table 2). Direct quantification of biofilm reduction showed that the mixed sugar solution had some activity against embedded biofilm cells of *S. epidermidis* and *C. albicans*, while only minor activity was seen for *S. aureus* and *P. aeruginosa* biofilms (Figure 1). MAWG solution at 80% (W/V) is acidic with a pH of ~4. A growth medium at pH = 4, but not the other pH values, showed some effects against biofilms pre-formed by *S. aureus*, *S. epidermidis*, and *C. albicans* (~30% inhibition; Figure 1). No significant effect of pH was found for biofilms formed by *P. aeruginosa* (Figure 1). None of the above-mentioned components of MAWG was able to eradicate biofilm cells of any of the four microorganisms, with MBECs beyond the highest concentration tested. Anti-biofilm activities of MGO in combination with other antimicrobial mechanisms were not studied due to little effect of MGO was observed against established biofilms.

### MAWG Has Minimal Activity on Biofilms Grown on the Exit-Site Wound Bed Mimics

**Figure 2:**
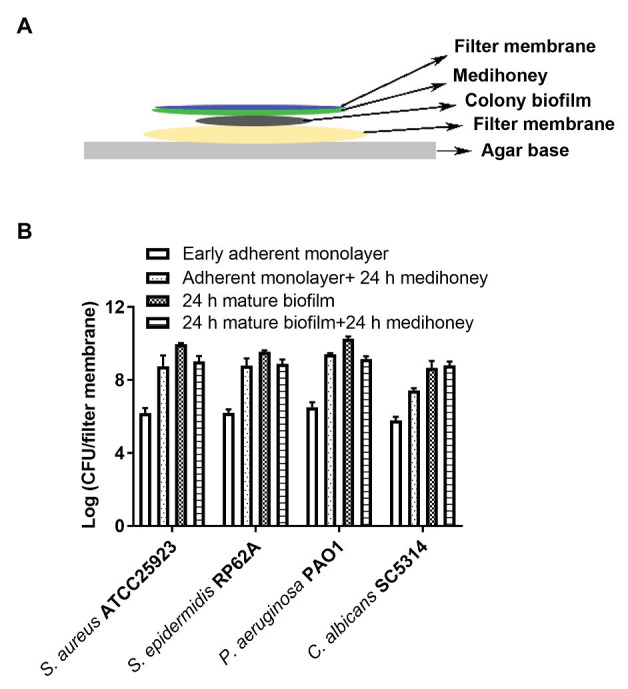
Assessment of activities of MAWG against colony biofilms at different developmental stages. **(A)** Schematic description of the colony biofilm assay. **(B)** Antibiofilm activities of MAWG against early adherent monolayer and mature biofilms. MAWG was found unable to effectively kill adherent monolayers; adherent monolayers developed into mature biofilms [2–5 log (CFU/cm^2^)] after overnight treatment. Only ~1 log reduction in CFU per colony biofilm was found when MAWG was used to challenge mature biofilms formed by *S. aureus*, *S. epidermidis*, and *P. aeruginosa*. No effect was observed for biofilms formed by *C. albicans*. Error bars indicate the standard error of the mean.

A colony biofilm assay in combination with MAWG-infused filter membrane (Figure 2A) was used to determine whether MAWG kills pre-established monolayers and biofilms mimicking those grown on the wound bed at the driveline exit-site, or interferes with the developmental process of biofilm formation. It was found that the MAWG-infused filter membrane had little effect on adherent monolayers of any of the tested microorganisms. All adherent monolayers grew into mature biofilms within a 24 h period on the agar plates and their biomass increased by 3 log (for *C. albicans*) or 4 log (for bacteria; Figure 2B). Biofilms of a high cellular density of ~10^9^ CFU/membrane (for bacteria) or ~10^7^ CFU/membrane (for *Candida*) were recovered (Figure 2B). For already established mature biofilms, MAWG only slightly reduced biomass of bacterial biofilms upon treatment, lowering the CFU by ~1 log (Figure 2B). No effect was observed for biofilms formed by the fungal pathogen *C. albicans* (Figure 2B).

### MAWG Has Minimal Activity Against Biofilms Growth on Drivelines at the Exit-Site

**Figure 3:**
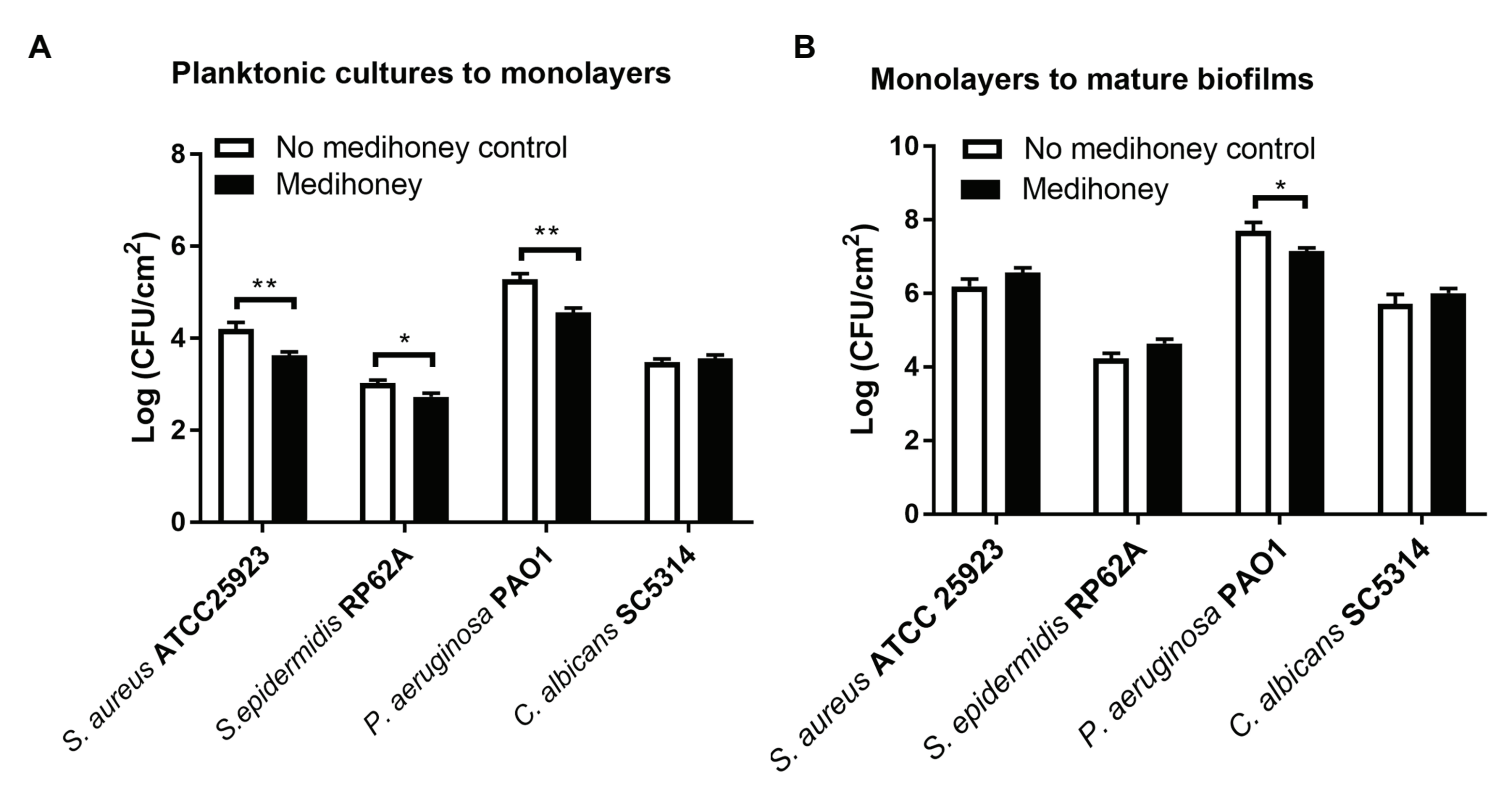
Determination of anti-biofilm activity of MAWG using a drip-flow biofilm reactor and driveline materials. **(A)** Adherent monolayers of microorganisms were formed on driveline smooth tube cut-outs pre-conditioned with MAWG. Driveline cut-outs received no treatment were used as a control. **(B)** Adherent monolayers formed on driveline cutouts (without MAWG conditioning) were transferred into a drip-flow biofilm reactor for further cultivation. Driveline cutouts with microbial monolayer were placed on absorbent pads infused with or without MAWG. Biofilm formation on driveline cutouts were assessed by viable counts after 72 h. Error bars indicate the standard error of the mean. ^*^p < 0.05; ^**^p < 0.01.

An early adherent monolayer assay and the drip-flow biofilm reactor assay were adopted to determine whether MAWG prevents planktonic cells from growing into adherent monolayers on the smooth tube section of drivelines and subsequently establishing biofilms. Covering driveline cutouts with MAWG lowered the biomass of adherent monolayers of *S. aureus*, *S. epidermidis* and *P. aeruginosa* by 12.9, 9.8, and 13.4%, respectively, as measured by viable counts (Figure 3A); a substantial number of adherent monolayers [2.7–4.6 log (CFU/cm^2^)] were stilled recovered from the MAWG-coated drivelines. Exposing adherent monolayers to MAWG in the drip-flow biofilm reactor did not result in a lower microbial density (log CFU/ml) of mature biofilms formed by *S. aureus*, *S. epidermidis*, and *C. albicans*, in comparison with the non-MAWG control (Figure 3B). One-log reduction in microbial density of mature biofilms was found when an adherent monolayer of *P. aeruginosa* was exposed to MAWG (Figure 3B).

## Discussion

Biofilm formation on percutaneous drivelines or surrounding tissues at the driveline exit-site is one of the most important factors contributing to the establishment of VAD-associated infections (Qu et al., 2020). This specific growth mode renders infections less responsive to antimicrobial treatments and is believed to be the root of the persistence of driveline infections (Qu et al., 2020). MAWG is routinely used for prophylaxis in all adult VAD programs in Australian hospitals. Despite the “excellent” *in vitro* anti-biofilm efficacy of Medihoney™ Antibacterial Honey, the main component of MAWG, reported by others (Muller et al., 2013; Cooper et al., 2014; Liu et al., 2017), driveline infections occurred frequently in VAD patients in these Australian hospitals. To clarify this discrepancy, a comprehensive assessment was conducted, using *in vitro* biofilm models that mimic the clinical environment of driveline exit-site. This study has shown that (1) MAWG inhibited and killed microorganisms under planktonic conditions in a species-specific manner, and had less inhibitory and microbicidal effect on biofilms pre-established in 96-well microplates, (2) the anti-biofilm activity of MAWG was mediated by multiple factors, with the often-advertised antimicrobial ingredient MGO only playing a minor role, and (3) under conditions that mimic the clinical environment, MAWG was neither able to kill monolayers or biofilms pre-established on tissue bed mimics, nor to prevent planktonic cells from growing mature biofilms on drivelines.

The driveline skin exit-site poses a huge risk of infections for VAD patients. Proper driveline exit-site care is considered effective in preventing infections during the healing period (Nienaber et al., 2013). Octenidine, dihydrochloride, chlorhexidine, and povidone-iodine have all been used to clean the skin around the driveline exit-site (Nienaber et al., 2013). Australian hospitals have often used 0.5% chlorhexidine in 70% alcohol solution to clean the driveline exit-site, followed by the application of MAWG as a prophylactic agent on the skin around the driveline exit-site and driveline dressing closure. While the use of prophylactic antibiotics has also been suggested peri-VAD implantation (Nienaber et al., 2013), no consensus has been reached regarding the optimal antimicrobial prophylaxis – either regime or duration (Walker et al., 2011; Nienaber et al., 2013). The prophylactic antimicrobial practice varies among different institutions and mostly relies on the team’s experience and preference (Nienaber et al., 2013). The general concept is to effectively cover common causative organisms of driveline infections, and to include agents for Gram-positive bacteria, Gram-negative bacteria, and fungi. The effectiveness of using conventional antibiotics in preventing driveline infections has been questioned (Stulak et al., 2013), and a major concern of the development of resistance to conventional antimicrobials has promoted the use of MAWG (Lu et al., 2014).

The broth microdilution method has been chosen by many others for the assessment of antimicrobial activity of medical-grade honeys against planktonic cells (Cooper et al., 2014; Osés et al., 2016). This assay yields more reproducible and informative results in comparison with other testing methods such as agar well diffusion or disk diffusion methods (Osés et al., 2016). Using the broth microdilution method, we also found potent species-specific antimicrobial activities of MAWG against planktonic cells. MAWG was highly effective against *S. epidermidis* and *S. aureus*, but not *P. aeruginosa* or *Candida* spp.. The role of individual components of honey in combating planktonic microorganisms has been extensively studied and seemed to be microorganism-specific (Kwakman and Zaat, 2012). Osato et al. (1999) found that the osmotic effect of honey was the most important mechanism for killing *Helicobacter pylori*, while hydrogen peroxide only played a minor role (Osato et al., 1999). Wasfi et al. (2016) studied Egyptian honeys and found their antibacterial activity against *Escherichia coli* was mostly caused by the production of hydrogen peroxide (Wasfi et al., 2016). Mavric et al. (2008) attributed the antibacterial potency of Manuka honey directly to the presence of MGO (Mavric et al., 2008). Using the microplate biofilm assay, we compared different components of MAWG that might contribute to its anti-biofilm potency and found that its anti-biofilm activities cannot be explained by one single antimicrobial mechanism. Unfavorable local environmental conditions resulted from the presence of honey including high-osmolarity and acidity, and other adverse effects occurring due to honey, such as desiccation (Park et al., 2016), might have hindered microbial biofilm growth. Notably, MGO, “the major antimicrobial effector” advertised by Comvita Ltd., failed to demonstrate a potent anti-biofilm effect in the current study. In support of our findings, Lu et al. (2014, 2019) reported that MGO in Manuka-type honey alone was inadequate in killing biofilms formed by *S. aureus* or *P. aeruginosa*.

In contrast to previous *in vitro* studies that reported outstanding anti-biofilm activities of Medihoney Antibacterial Honey (Muller et al., 2013; Cooper et al., 2014; Liu et al., 2017), the high-quality randomized controlled Honeypot trial recently found no superiority of MAWG in preventing biofilm-related peritoneal-dialysis-related infections or exit-site infections when compared with standard care (Johnson et al., 2014; Zhang et al., 2015). We speculated that the anti-biofilm effectiveness reported by other *in vitro* studies might be overly optimistic, partially due to the use of *in vitro* assays with minimum clinical relevance, such as the microplate-based biofilm assay (Lu et al., 2014; Buhmann et al., 2016; Liu et al., 2017; Piotrowski et al., 2017). Biofilms grown in 96-well microplate might not adequately reflect those found in a more complicated scenario such as driveline infections, where biofilms were often grown at a liquid-solid-air interface with low shear force and moisture. Our study used *in vitro* models that closely mimic the clinical environment of the driveline exit-site and evaluated MAWG as a therapeutic agent and a prophylactic agent for driveline infections, respectively. The colony biofilm assay showed that MAWG-infused filter membranes that mimic topical dressings with MAWG impregnated were unable to inhibit or kill pre-established adherent monolayers or biofilms, questioning the value of MAWG in treating driveline exit-site wounds. Our drip-flow biofilm reactor assay also found limited efficacy of MAWG in preventing biofilm formation on drivelines, supporting the conclusion from the large-scale Honeypot clinical trial (Zhang et al., 2015). We noticed differences in the anti-biofilm efficacy of MAWG against *P. aeruginosa* PAO1 when microplate-based biofilm assay and the drip-flow biofilm assay reactor assay were carried out. This can be explained by different environmental factors of these two biofilm assays that might affect the anti-biofilm activity of MAWG (Buhmann et al., 2016). We concede that two non-microplate-based *in vitro* biofilm assays chosen for this study could not completely duplicate the complexity of the clinical environment, but we managed to include some environmental factors such as local oxygen availability and low shear at the air-liquid-solid interface that are critical for biofilm development at the driveline exit-site or important for the assessment of anti-biofilm agents. A large-scale randomized-controlled clinical trial will facilitate a better understanding of the effectiveness of MAWG in preventing driveline infections in VAD patients.

## Conclusion

Taken together, our data showed little success of MAWG in killing biofilms grown on tissue bed mimics, or in preventing biofilm formation on driveline materials, suggesting suboptimal effectiveness of MAWG as a therapeutic or prophylactic agent against biofilm-related driveline infections. Though routine application of MAWG might partially prevent microbial contamination of the driveline exit-site, supported by its effectiveness against planktonic microorganisms, caution must be exercised when relying on MAWG to prevent or treat driveline infections in patients with a VAD.

## Data Availability Statement

The raw data supporting the conclusions of this article will be made available by the authors, without undue reservation.

## Author Contributions

YQ, DM, and AP conceived and designed the study. YQ carried out the experiments. YQ, CK, JM, and CD performed data analysis. YQ and DM wrote up the manuscript. AP edited the manuscript. All authors contributed to the article and approved the submitted version.

### Conflict of Interest

DM is an Abbott proctor for implantation of the Heartmate III VAD.

The remaining authors declare that the research was conducted in the absence of any commercial or financial relationships that could be construed as a potential conflict of interest.
